# CO_2_ Laser Fabrication of PMMA Microfluidic Double T-Junction Device with Modified Inlet-Angle for Cost-Effective PCR Application

**DOI:** 10.3390/mi10100678

**Published:** 2019-10-09

**Authors:** Gamal A. Nasser, Ahmed M.R. Fath El-Bab, Ahmed L. Abdel-Mawgood, Hisham Mohamed, Abdelatty M. Saleh

**Affiliations:** 1 Mechatronics and Robotics Department, Egypt-Japan University of Science and Technology (E-JUST), Alexandria 21934, Egypt; ahmed.rashad@ejust.edu.eg; 2 Mechanical Engineering Department, Assiut University, Assiut 71515, Egypt; 3 Biotechnology Department, Egypt-Japan University of Science and Technology (E-JUST), Alexandria 21934, Egypt; almawgood@yahoo.com; 4 Institute of Graduate Studies and Environmental Research, Damanhour University, Damanhour 22511, Egypt; 5 Biomedical Engineering Department, Rensselaer Polytechnic Institute, Troy, NY 12180, USA; mohamh2@rpi.edu; 6 Fish Processing and Biotechnology Department, Faculty of Aquatic and Fisheries Sciences, Kafrelsheikh University, Kafr-Elsheikh 33516, Egypt; abdelattysaleh@fsh.kfs.edu.eg

**Keywords:** droplet formation, PMMA, microchannel, ddPCR, CO_2_ laser micromachining

## Abstract

The formation of uniform droplets and the control of their size, shape and monodispersity are of utmost importance in droplet-based microfluidic systems. The size of the droplets is precisely tuned by the channel geometry, the surface interfacial tension, the shear force and fluid velocity. In addition, the fabrication technique and selection of materials are essential to reduce the fabrication cost and time. In this paper, for reducing the fabrication cost Polymethyl methacrylate (PMMA) sheet is used with direct write laser technique by VERSA CO_2_ laser VLS3.5. This laser writing technique gives minimum channel width of about 160 μm, which limit miniaturizing the droplet. To overcome this, modification on double T-junction (DTJ) channel geometry has been done by modifying the channel inlets angles. First, a two-dimensional (2D) simulation has been done to study the effect of the new channel geometry modification on droplet size, droplets distribution inside the channel, and its throughput. The fabricated modified DTJ gives the minimum droplet diameter of 39±2 μm, while DTJ channel produced droplet diameter of 48±4 μm at the same conditions. Moreover, the modified double T-junction (MDTJ) decreases the variation in droplets diameter at the same flow rates by 4.5–13% than DTJ. This low variation in the droplet diameter is suitable for repeatability of the DNA detection results. The MDTJ also enhanced the droplet generation frequency by 8–25% more than the DTJ channel. The uniformity of droplet distribution inside the channel was enhanced by 3–20% compared to the DTJ channel geometry. This fabrication technique eliminates the need for a photomask and cleanroom environment in addition shortening the cost and time. It takes only 20 min for fabrication. The minimum generated droplet diameter is within 40 μm with more than 1000 droplets per second (at 10 mL/h. oil flow rate). The device is a high-throughput and low-cost micro-droplet formation aimed to be as a front-end to a dynamic droplet digital PCR (ddPCR) platform for use in resource-limited environment.

## 1. Introduction

Droplet-based polymerase chain reaction (dPCR) enables a more precise, sensitive and reproducible target quantification compared to conventional (PCR). In dPCR, nucleic acid sample is partitioned into a mass of compartments (e.g., droplets and microwells) or chambers [1]. The PCR method is extensively utilized as a molecular biological tool for DNA amplification, and makes replicas of specific fragments of DNA over three temperature cycles. Studying DNA requires analyzing hundreds of samples to have enough statistics to draw a useful conclusion. Great effort and time is spent on optimizing these tests to come up with a robust procedure, which by itself requires more tests and costs. The combination of the high cost and the need to analyze hundreds of samples for a single study is obstructing progress in research and in clinical applications [2,3]. Moreover, in some applications, in which the subjects are of a low copy number in a large background such as cancer, prenatal analysis, and genetically modified organisms (GMO), high sensitivity of detection is needed. As in those applications if the mutation is less than 3%, it cannot be detected by traditional PCR [4,5]. This combination problem could be solved by introducing a low-cost micro-droplet formation chip to make the sample separate into a large number of small partitions and the reaction is carried out in each partition individually [6,7,8,9]. This separation allows a more reliable collection and sensitive quantification of nucleic acid signals [10]. These special behaviors can improve efficiency and decrease cost of reagents and chemical waste [11,12,13].

Droplet formation using microfluidic channel for dynamic continuous-flow-based PCR device has been done using three methods as follows: (1) co-flowing dripping streams, (2) cross-flowing dripping streams and (3) flow focusing dripping streams [14,15]. Although the effect of shear forces, interfacial forces and channel dimensions on droplet generation have already been widely studied, emulsification using cross-flow microfluidics devices is considered quite new, [16,17,18]. Moreover, cross-flow is considered the most popular emulsification method [19]. Many methods are used for channel manufacturing in cross-flow streams. The first technique is “microfabrication” especially soft lithography technique which is a mix of additive (deposition) and subtractive (etching) techniques at micro or submicron scale. Soft lithography technique is one of the most popular techniques to make biomedical microfluidic system. However, the silicon mold fabrication to create microfluidic chips, generally needs a cleanroom environment, photolithography machine, photomask, expensive infrastructures, and long development time from design to device [17,20,21,22,23]. Consequently, numerous efforts have been made to improve low-cost alternatives for microstructures fabrication, which avoids usage of cleanroom facilities. The second technique is “Subtractive manufacturing”, which requires regular machining such as using a milling machine to remove materials [24]. In this case, the cross section of the channel is rectangular but there are several drawbacks, for instance complex tool alignment [17]. In addition, mechanical micro-milling machine needs a highly precise CNC system with corresponding tool library [25]. The third technique is “Additive manufacturing” such as 3D printing, which is a layer-by-layer manufacturing process and has been extensively used in several fields such as aerospace, organ printing and industrial design. In the last ten years, the 3D printed microfluidics method has seen growing interest because of its fast printing in the lab [26,27]. However, this method has limitations such as feature size, rough surface and low resolution in addition to the lack of material availability.

Another fast prototyping technique is direct write laser method such as CO_2_ laser, which has many advantages such as low cost, short prototyping time, and no cleanroom facilities or chemicals are required [28]. However, this technique produces non-rectangular cross section channel with random surface roughness [29,30]. Laser engraving is between subtractive technique (uses laser instead of a cutting tool) and micro fabrication (similar scale without the mask and clean room facility). In recent years, many researchers have used direct write laser technique, with different materials such as silicon [31], glass [32,33,34], and PDMS [23,35,36]. Recently, microfluidics researchers give more interest to Polymethyl methacrylate (PMMA) material [30,37,38,39,40], as it is a thermoplastic material with admirable biocompatibility, durability, transparency, non-porosity, low-cost and availability. PMMA is not easy to cast because of its elastomeric nature [19]; however, PMMA can be engraved and cut easily with a CO_2_ laser [30]. Some researchers provided a hybrid method between laser and chemical etching to produce a circular channel [41,42]. Prakash et al. used a copper mask on PMMA substrate under water to generate rectangular cross-sectional micro-channels [43]. Another modification is using a thin layer of water above PMMA to reduce the heat affected zone and avoid clogging formation. This leads to high aspect ratio by using laser under water [44,45,46,47]. However, all of these hybrid methods need additional tools and facilities, thus the fabrication cost and time is increased. After the channel fabrication, the channel is covered with another layer by bonding. Many bonding processes have been provided to cover the channel, such as solvent bonding [48], adhesive bonding [49], and thermal bonding [50,51,52].

There are three main factors that manage the droplet formation process and breakup dynamics in cross-flow streams; the first factor is channel design, (i.e., channel dimensions, geometry and hydrophobicity), the second is fluid properties (i.e., viscosity, density, contact angle and interfacial tension), and the third is operating parameters (i.e., pressure, temperature, flow rate ratio, etc.) [53].

From the geometry point of view, the droplet formation is started by inlet channel with T-junction shape [54]. Then the DTJ channel is used to give high sheer force [55] which results in low droplet size with high droplet generation frequency. It is only in recent times that several researchers studied the role of the inlet geometry, mainly the effect of inlet angle. Researchers revealed that the droplet formation process is affected significantly by inlet geometry design [53,55,56]. Inlet geometry angle has an essential role in the cross region dynamics in the squeezing regime. Therefore, it has obvious effect of the generated droplet’s size. This angle has more influence in the dripping regime at 30° than 90° on drag and viscous shear which acting on the forepart of the dispersed flow. As a result, the droplet formation cycle is reduced more considerably, and speeds up the necking process [56]. In a cross-junction alternating regime device, droplet formation is more efficient at 45° angle of the two side channels relative to the main channel [57] thus the two side channels have 90° in between. 

In this paper, a micro-droplet formation chip for dynamic continuous-flow-based PCR is fabricated by direct write laser technique on PMMA material. This cheap combination between low cost of fabrication and raw material produces a micro-droplet formation chip for less than 30 cents. As this fabrication technique produced relatively wide channel (about 160 μm minimum), which limit miniaturization of the droplets, we introduced a new inlet channel geometry which may produce small droplet size relative to the channel size. The new inlet channel geometry is called modified double T-junction (MDTJ). The performance of the two channel geometries, namely the double T-junction (DTJ) and the modified double T-junction (MDTJ) were compared regarding droplets parameters (generated droplet size, droplet generation frequency and droplets separation distance). Finally, the fabrication repeatability is discussed to determine how far this fabrication method is suitable for mass production.

## 2. Materials and Methods

### 2.1. Device Description and Design Modification

**Figure 1:**
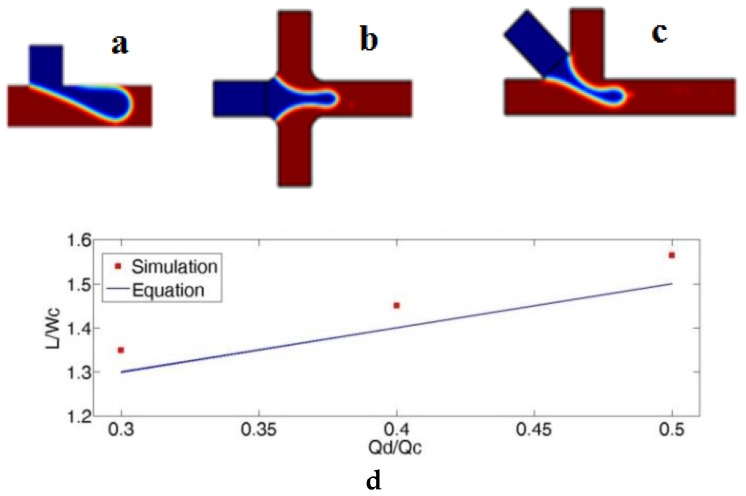
(**a**–**c**) photos show droplet break up region for the three channels geometry which recorded from simulation as follows: (**a**) Traditional T-Junction; (**b**) double T-junction (DTJ) channel; (**c**) modified double T-junction (MDTJ) channel. Blue is water and red is oil. (**d**) This graph illustrates the error between simulated T-Junction channel and Equation (8).

Normally, the confined T-junction channel has one inlet for paraffin oil (red) and another inlet for PCR mix (blue) (Figure 1a). In the DTJ channel there are two channels perpendicular on the main channel for paraffin oil 180° in between and the other channel inlet for PCR mix (Figure 1b). Here, in order to facilitate visualization, a blue dye solution was used as a sample [58]. By considering the effect of inlets angle from the previous studies as mentioned before and combining DTJ with confined T-junction channel we got our new inlet geometry. The modification is the PCR mix input was made at 45° between the two oil inlets (Figure 1c) and the two side oil channels have 90° in between. The modification could accelerate the necking process in the dripping regime. The forepart of the dispersed flow thread at angle lower than 90° is greater than that under angle equal to 90°. The main reason behind that effect is due to the lower squeezing action from the continuous flow; once the pinch-off and necking of the dispersed flow move downstream at the cross region, it is fundamentally more influenced by the drag and viscous shear from the continuous flow than the conventional geometry. Accordingly, the droplet formation cycle is reduced to generate smaller droplets and higher frequency. Therefore, we refer to this geometry modification as modified double T-junction (MDTJ).

### 2.2. Governing Equations

The microfluidic devices have laminar fluid flows because of low Reynolds number [59]. Therefore, the Navier–Stokes (1), volume fraction (2), and continuity Equations (3) can describe these fluid flows [60]. Using level-set method, which is provided by Reference [61], to simulate two immiscible fluids flow separated by moving interface.

(1) $$\rho \frac{\partial \vec{u}}{\partial t}+\rho \left(\vec{u}\cdot \nabla \right)\vec{u}=\nabla \cdot \left[-PI+\mu \left(\nabla \vec{u}+\nabla {\vec{u}}^{T}\right)\right]+\vec{F}+\rho \vec{g}+\sigma k\delta \vec{n}$$

(2) $$\frac{\partial \varphi }{\partial t}+u\cdot \nabla \varphi =0$$

(3) $$\nabla \cdot \vec{u}=0$$

where ρ, u, P and μ are the density, flow velocity, static pressure, and dynamic viscosity of the fluid respectively. The left side term of Equation (1) is a velocity derivative with respect to the time t. I is the unity matrix, $\vec{F}$ are any other forces acting on the fluid which called body forces and the term $\sigma k\delta \vec{n}$ in the right side of Equation (1) is the force of volumetric surface tension acting on the interfaces between the two fluids [62], where k, σ and $\vec{n}$ are curvature, the coefficient of surface tension, and unit surface normal of the interface respectively. δ is Dirac delta function, which is zero anywhere except at the interface. φ is volume fraction, which changes sharply from 0 to 1 at the interface [63]. These parameters were specified in Equation (4).

(4) $$\vec{n}=\frac{\nabla \varphi }{\left|\nabla \varphi \right|},\;k=\nabla \vec{n},\;\delta =6\left|\nabla \varphi \right|\left|\varphi \left(1+\varphi \right)\right|$$

In Equation (5), the left side defines the interface motion and the right-hand side represents numerical re-initialization and stabilization, where ε is interface thickness controlling parameter and γ is intensity re-initialization parameter.

(5) $$\frac{\partial \varphi }{\partial t}+u\cdot \nabla \varphi =\gamma \nabla \cdot \left(\varepsilon \nabla \varphi -\varphi \left(1-\varphi \right)\frac{\nabla \varphi }{\left|\nabla \varphi \right|}\right)$$

In this case the dynamic viscosity and the density are described in Equations (6) and (7).

(6) $$\mu =\mu_{1}+\left(\mu_{2}-\mu_{1}\right)\varphi$$

(7) $$\rho =\rho_{1}+\left(\rho_{2}-\rho_{1}\right)\varphi$$

where $\mu_{1}$, $\mu_{2}$ are the dynamic viscosities and $\rho_{1}$, $\rho_{2}$ are the densities of oil and water, respectively. The flow conditions at inlets are laminar flow and normal inflow velocity. Additionally, a zero pressure exists at the boundary in outflow and the channel walls are no-slip walls.

### 2.3. Simulation Model Verification

**Figure 2:**
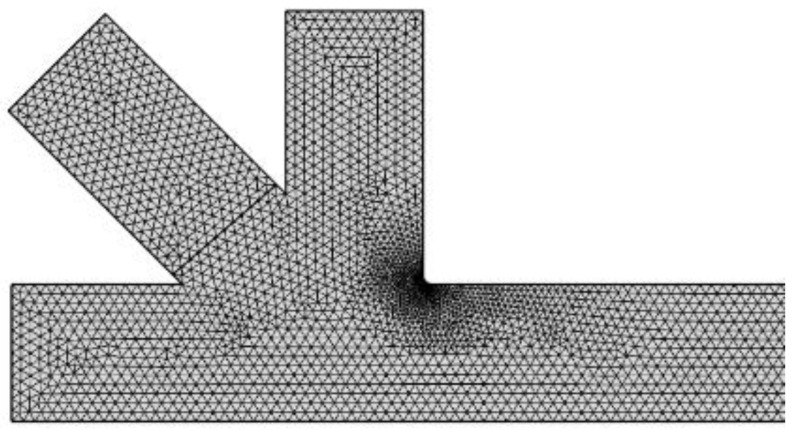
Created mesh for a crossed part of the channel.

In this section, our finite element model needs to be verified before using it to compare between the DTJ and MDTJ. Therefore, Equation (8) presents a simple dimensionless equation that describes the length of plugs formed in T-junctions [64]. A recent research in Reference [65] utilized this equation to compare with the experimental data. The experiments were conducted for different fluid flow rate combinations in a T-junction, where all branches had internal diameters equal to 200 μm. The dispersed phase was a water/glycerol solution and was injected from the side branch of the junction, while the continuous phase was silicone oil and was injected along the main channel axis. The width and depth channel of the continuous and dispersed phase are comparable or equal to one another (that is confined droplet breakup).

(8) $$\bar{L}=L/w_{c}\;=1+\alpha \phi$$

where α is a constant factor related to the width ratio of the dispersed and continuous phase channels, $w_{c}$ is the width of the continuous phase channel, ϕ is the flow rate ratio, and L is the plug length. Therefore, a 2D simulation for T-junction was done with the same dimensions, fluid properties, and flow rates to check the model with Equation (8). The simulation gave the same trend of this equation with maximum error of 4.3% as shown in Figure 1. However, to minimize the calculation time for the proposed geometry the simulation was concentrated on the breakup region as shown in Figure 2. For this reason, the entrance distance is 300 μm for the three inlet channels and 5000 μm for the outlet channel. The element shape is equilateral triangle with minimum and maximum rib length of 3 μm and 10 μm respectively.

The channel dimension is one of the important parameters, which directly affect the generated droplets size. Experimentally, a laser cutting machine was used to create the channel in PMMA substrate, which gives a surface channel width of about 159 μm and depth of about 105 μm after bonding; with channel roughness ranging from Ra=3.2—6 μm. Therefore, these dimensions were used in the simulation (measured using 3D laser microscope KEYENCE VK-X100 (Keyence Corporation of America, Elmwood Park, NJ, USA)).

### 2.4. Channel Fabrication and Dimension Repeatability

**Figure 3:**
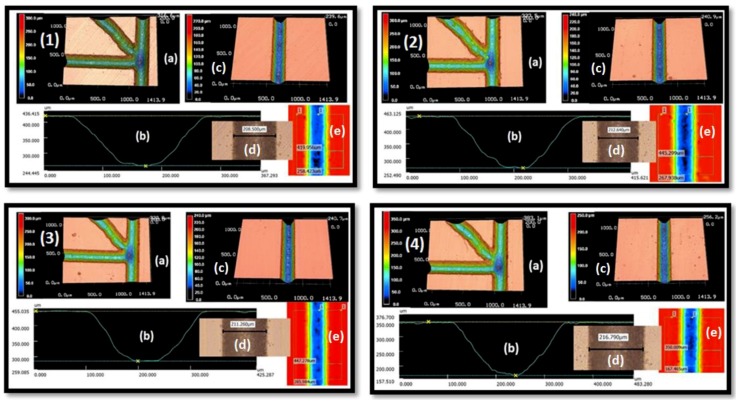
Repeatability of the manufacturing. Four different manufacturing samples (1–4). (**a**) 3D projection of the channel at the cross region, (**b**) Channel profile, (**c**) 3D projection of the main channel, (**d**) surface width of the channel and (**e**) colored photo to show width and depth. Sample 1 surface width 208 μm, Sample 2: surface width 212 μm, Sample 3: surface width 211 μm and Sample 4: surface width 216 μm.

In this section, four different samples are fabricated with the same condition to check the repeatability of the produced channel by direct write laser machining technique. VLS3.5 UNEVERSAL LASER SYSTEMS with a 30-watt CO_2_ laser tube and 100 μm laser beam diameter was used for channel fabrication. The chip consists of 2 PMMA parts, the lower part contains the engraved channel structure, and the upper part contains inlet ports. We got the best engraving by adjusting the engraving speed to $25\;\mathrm{mm}.s^{-1}$ (10%) laser head translation speed and laser beam power to 5 watt (6%) laser beam power to get less roughness at lowest available dimensions. The channel profile is Gaussian shape as shown in Figure 3b.

The channel was characterized using the 3D laser microscope KEYENCE VK-X100. This microscope can measure the channel profile, depth, width and roughness as shown in Figure 3. The figure represents four individual photos for the four tested samples, each photo contains five detailed photos for each sample. The surface width of the fabricated channels was about 212 ± 4 μm. The imaging of the four samples shows that all channels are open and clean enough to have unblocked flow.

### 2.5. Chip Bonding and Its Final Dimension

The bonding of the top part (which contains the inlets and outlet holes/ports) and the bottom part (which contains the channel) was done by thermocompression method with acetic acid at 120 °C and 1.5 N for 10 min. By heating with acetic acid, better bonding at lower temperature was achieved, as well as better bonding time [66].

**Figure 4:**
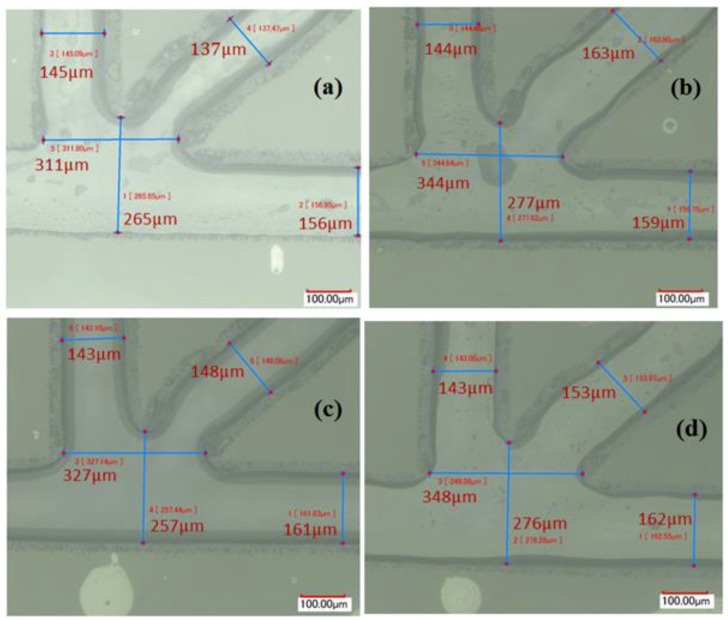
Shows channel dimension after bonding for the 4 samples with scale bar 100 μm, Water was injected to show the boundaries of the channel, then air to make a good appearance of the channel boundary. The order of the four samples is as follows: (**a**) first sample; (**b**) second sample; (**c**) third sample; (**d**) fourth sample.

Therefore, the width at the surface of the channel reduced approximately from 210 μm to 159 μm. This is due to the compressive force applied to the top and bottom parts during the thermal bonding process. Fortunately, this phenomenon is useful as we generally seek smaller dimension to achieve smaller droplet. These measurements have been done after channel leakage test by water injection. In order to show the repeatability of the channel dimension after bonding, the channel width is recorded for the 4 samples (Figure 4). The horizontal and vertical channels dimensions are repeatable with deviation of about ±2 μm, representing an error of approximate ±1.5%. The inclined channel shows a relatively remarkable dimension deviation of about ±12 μm which amount to an approximate error of ±8%.

### 2.6. Experimental Setup

**Figure 5:**
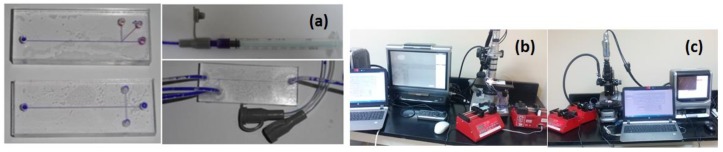
Illustrates experimental setup: (**a**) channels after bonding, connecting tubes and connectors. The channel was attached to a 1 mm syringe which contains water (blue). (**b**) Digital microscope, (**c**) High speed microscope. For ease changing flow rate, programmable syringe pumps controlled by the LabVIEW program were used.

The experimental setup consists of two syringe pumps DUAL-NE-1000X (New Era Pump Systems Inc., Farmingdale, NY, USA); one single syringe for water flow and the other is a double syringe for oil flow. One 1 mL syringe for water and two 3 mL syringe for oil were attached to the chip using Infant type feeding tubes by cyanoacrylate based chemical bonding as shown in Figure 5a. The device sealing was tested by flowing water through the channel under relatively high flow rate to figure out any leakage and test the channels for droplet generation. The digital microscope KEYENCE VHX-1000 which attached with KEYENCE VH-Z100R wide range zoom lens (100–1000×) (Keyence Corporation of America, Elmwood Park, NJ, USA) was used for measuring droplets diameter and the distance between them (Figure 5b). High speed microscope KEYENCE VW-9000 was used for droplet formation frequency measurements (Figure 5c).

## 3. Results and Discussion

### 3.1. Simulation Results

In this section, the effect of the ratio of oil-to-water flow rates on the droplet parameters (diameter, frequency, and separation distance) for the two channels geometries (DTJ and MDTJ) is presented using 2D finite element model. Light liquid paraffin oil was used as the continuous phase while blue colored water as the dispersed phase with the following properties; Water: density $\rho =1020\;\mathrm{kg}/m^{3}$ and viscosity μ=0.001 Pa·s and, oil: density $\rho =855\;\mathrm{kg}/m^{3}$ and viscosity μ=0.075 Pa·s. The interfacial tension between the two immiscible liquids is σ=0.05 N/m [67]. With the fluids flow rates of water kept fixed at 0.3 mL/h, while paraffin oil flow rate varied from 0.3 mL/h to 7 mL/h.

**Figure 6:**
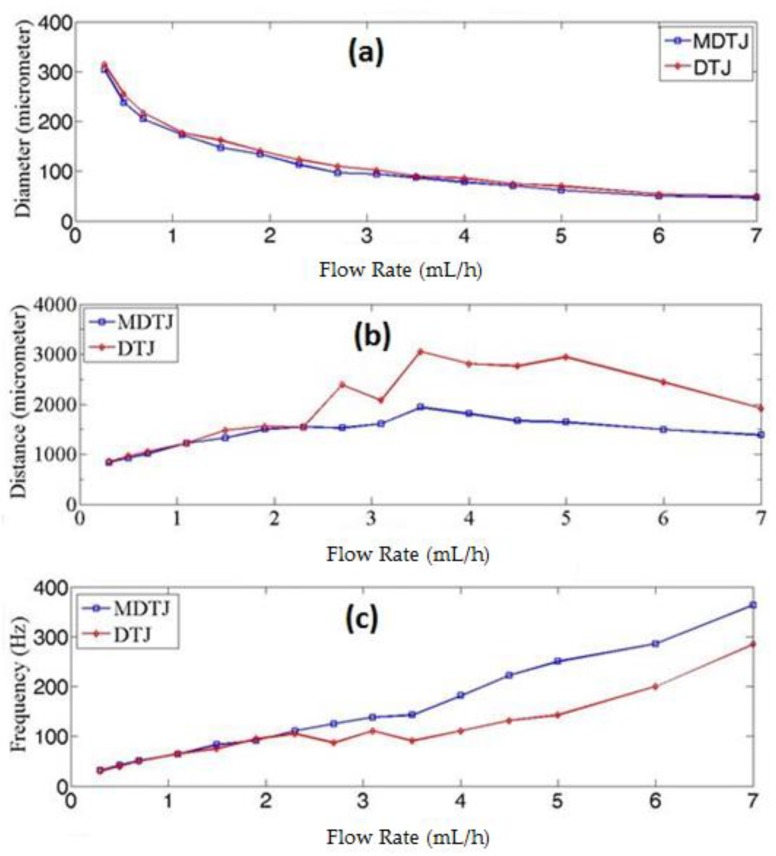
Three different parameters for the two geometries comparison is illustrated as follows: (**a**) droplet diameter (**b**) distance between droplets and (**c**) droplet frequency with varied oil flow rates ranged from 0.3 mL/h to 7 mL/h and fixed water flow rate at 0.3 mL/h.

The results of simulation presented in Figure 6 shows that the MDTJ has great effect on droplet generation. Although at very low flow rate plugs were generated, with increased oil flow rate the droplets start to form. The droplet length ranged from 304 μm at 0.3 mL/h to 43 μm at 7 mL/h oil flow rate in the MDTJ channel. This gives us the ability to produce Nano-Liter to Pico-Liter droplets without the need of expensive clean room facilities. Using the same condition, the DTJ channel produces droplets from 314 μm to 54 μm (Figure 6a). The second important parameter in droplet generation is the distance between droplets. This distance should be uniform when oil and water are at fixed flow rate, which is important in the future for DNA fluorescence detection. Figure 6b illustrates this distance with changing oil flow rate for the two channels geometries. When the oil flow rate changed from 0.3 to 2.3 mL/h the two channels gave almost the same trend. However, after further increasing the oil flow rate, the DTJ channel had some fluctuation in this distance. On the other hand, the MDTJ channel maintained small fluctuation in the distance between droplets. At 3.5 mL/h oil flow rate, the maximum distance between droplets was 1.937 mm in the MDTJ channel and 3.055 mm in the DTJ channel. The third important parameter is the droplet frequency shown in Figure 6c, which is the number of generated droplet in one second. At low oil flow rate (0.3–2.3 mL/h) the two channels generated almost the same number of droplets. However, when the oil flow rate increased the MDTJ channel produced more droplets than the DTJ channel.

### 3.2. Experimental Results

**Figure 7:**
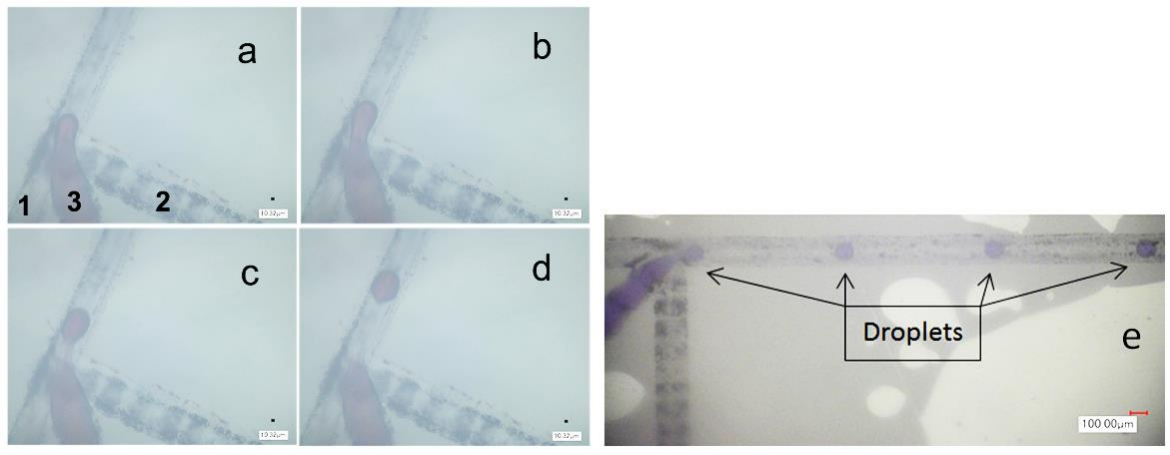
Droplet breakup in the MDTJ from (**a**) to (**d**) (this channel belongs to channel in Figure 4a) Channels 1 and 2 are oil (1.1 mL/h) and Channel 3 is the water at 0.3 mL/h. (**e**) Real photo to illustrate the uniformity of droplet distribution inside the channel at 3 mL/h oil flow rate and 0.3 mL/h water flow rate with scale bar 100 μm.

A higher oil flow rate (above 1.1 mL/h) was used in this study because, as stated earlier, at low flow rate plugs are generated. Similarly, at low water flow rate (below 0.3 mL/h) there is fluctuation in the droplet, i.e., pulsing flow, this is due to low speed of the pump’s stepper motor. Figure 7 illustrates the modified channel breakup region: oil enters through channel Inlets 1 and 2 while channel Inlet 3 has the colored water. Additionally, Figure 7e provides visual impression for the uniformity of droplet distribution inside the channel.

**Figure 8:**
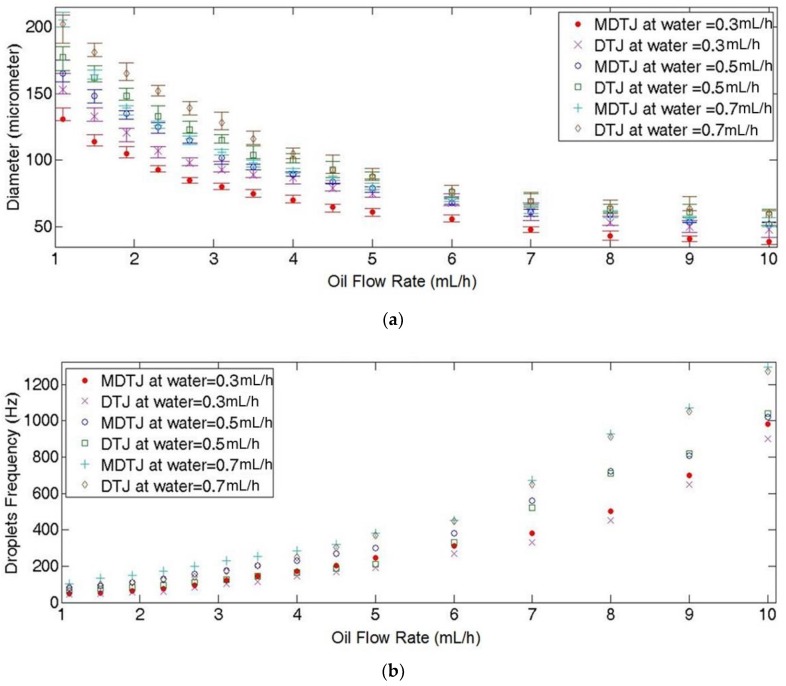

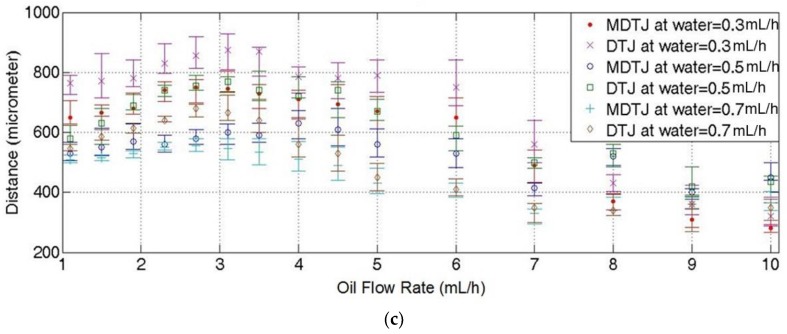
Illustrates three different parameters for the two channel geometries to characterize MDTJ channel. These results were measured at different oil flow rate ranging from 1.1 to 10 mL/h at water flow rates of 0.3 mL/h, 0.5 mL/h and 0.7 mL/h, as follows: (**a**) Droplet diameter which was measured from 19 readings. (**b**) Droplet frequency which was manually counted from high speed microscope video. (**c**) Distance between droplets inside the two channels which was measured from 17 readings. Error bar in (**a**) represents the variation of droplets size and in (**c**) represents the variation of interspacing distance between droplets.

At fixed water flow rate, as the oil flow rate increases the droplet diameter decreases, as presented in Figure 8a. In this figure, the MDTJ generates smaller droplets and closer to each other than the DTJ at the same conditions. Additionally, by using MDTJ the minimum droplet diameter of 39±2 μm was found at both maximum oil flow rate and minimum water flow rate (±5.1%) while DTJ channel produced 48±4 μm at the same conditions (with 8.3% error). However, these experimental errors decreased at the middle oil flow rate region. For instance, at 4 mL/h the MDTJ generated droplets diameter of 70±2 μm (±2.8%), while at the DTJ channel the droplet diameter was 85±4 μm (±4.7%). Increasing the water flow rate, led to increase in the droplet diameter at all range of oil flow rate. Generally, the MDTJ channel decreases the variation between droplet diameters by maximum of 9.7% than the DTJ channel.

Figure 8b represents droplet frequency and illustrates the difference between the two channels in this parameter. Firstly, at 0.3 mL/h water flow rate and low oil flow rate (1.1–2.3 mL/h), both MDTJ and DTJ channels produced almost the same number of droplets per second. However, with increasing oil flow rate above 2 mL/h, there is a difference in the number of droplets per second between the two channels. The MDTJ channel produces 8–25% higher number of droplets than the DTJ. However, by increasing the water flow rate from 0.3 mL/h to 0.5 mL/h the maximum difference between the two channels geometries was found at the mid-range of oil flow rate. Additionally, when the water flow rate reached 0.7 mL/h the maximum difference crawls to low oil flow rate region. Generally speaking, the MDTJ channel has low droplet-breakup time than the DTJ channel.

Uniformity of distribution of the droplets in both MDTJ and DTJ were measured by recording 17 different readings to make a statistical conclusion. Figure 8c illustrates distribution of the droplets and shows that the MDTJ channel has minimum distance and distance variation between droplets within the channel. At water flow rate of 0.3 mL/h the MDTJ channel produced the minimum distance between the droplets at any oil flow rate in the range of 1.1–10 mL/h. The distance between droplets inside the MDTJ channel is less than DTJ channel. For instance, a difference of 40 μm (12.5%) at 10 mL/h oil flow rate, and 140 μm (16%) at 3.5 mL/h oil flow rate. However, at 6 mL/h oil flow rate, the distance between droplets had largest variation of up to 10%. This variation was noticed in both channel geometries. But, after increasing water flow rate from 0.3 mL/h to 0.5 mL/h, the largest difference in droplet distance between the two channels started from oil flow rate of 1.1 mL/h to 6 mL/h peaking at 2.3 mL/h. At this peak, the MDTJ generated droplet distance less than DTJ by 180±30 μm (24±4%). The highest variation in droplet distance of about 10.25% was found at oil flow rate of 4.5 mL/h in both channel geometries. By increasing the water flow rate to 0.7 mL/h the maximum difference in droplet distance between the two channels crawls to low oil flow rate region and the MDTJ channel got the least deviation. However, the two channels recorded the largest deviation in the middle oil flow rate region ranging from 4 mL/h to 5 mL/h. In general, changing water flow rate has more effect on distance between droplets at low oil flow rate. While by increasing oil flow rate, the distance and distance variation between droplets decreased for both channel geometries.

Our results show that MDTJ has several advantages over the DTJ, i.e., smaller droplets size, larger number of droplets per second and higher uniformity in droplets distribution. Table 1 shows the comparison between results obtained from the simulation and the experiment at the same oil and water flow rates. There is difference between simulation and experimental in all three parameters. For example, in the experimental, both MDTJ and DTJ produced lower droplet diameter than simulation. This is also true for the other parameters. Although there is difference between simulation and experimental results, the trend is similar by comparing Figure 6 and Figure 8. The difference is due to using 2D simulation model [15], the actual channel is not rectangular profile as in simulation and the surface roughness is not identical. In Table 2 we summarize a comparison between the work presented here and other works under almost similar conditions. The MDTJ produced smaller droplet diameter than the previous reports [15,19,30,39]. Islam et al. used 6 mL/h oil flow rate and 0.3 mL/h water flow rate to produce 60 μm droplet diameter, however, at the same conditions the MDTJ produced 54 μm and the DTJ generated 66 μm. We only compared results at the same flow rates and dimensions. However, Islam et al. achieved smaller droplets but by reducing water flow rate than 0.3 mL/h [19]. The minimum flow rate was achieved is 0.3 mL/h by our existed syringe pump because lower than this flow rate, the pump generated pulsatile flow due to low speed of stepping motor, therefore the flow did not move smoothly. Yu et al. used a micromold fabrication method for PMMA microfluidic devices in addition they provided low temperature and deformation-free bonding method for channel covering. They needed more time for fabrication and covering the channel (almost one day) [39]. On the other hand, CO_2_ laser technique is the best method in terms of consumed time at fabrication process as well as its cost because this technique does not require chemicals (for etching) or expensive equipment. Among them our device is the least in fabrication cost, which cost less than 30 cents per device as shown in Table 2. The ground of cost estimation of our work is raw material and machining costs. Although, all these advantages of CO_2_ laser fabrication technique, it has low fabrication accuracy than the other expensive techniques (Photolithography, micromold, etc.)

## 4. Conclusions

A micro fluidics chip for micro-droplet formation was fabricated at a very low cost (less than 30 cents). The fabrication was done by a universal laser direct writing machine and using PMMA material as substrate. This combination (low-cost fabrication + low-cost material) makes the chip a good candidate for single-use in medical analysis. The effect of the channel geometry of the inlet channels intersection, namely DTJ and MDTJ, on the droplet size, droplet frequency, and distance between droplets at different oil and water flow rates has been studied.

The minimum droplet diameter (39 μm) was achieved by MDTJ at 10 mL/h oil flow rate and 0.3 mL/h water flow rate which meet the picoliter volume of droplets required for digital PCR device. The MDTJ gave smaller droplet size as well as more homogenous droplet diameter than DTJ. The MDTJ decreases the variation in droplets diameter at the same flow rates by 4.5–13% than DTJ. This low variation in the droplet diameter is suitable for repeatability of the DNA detection results. The MDTJ also enhanced the droplet generation frequency by 8–25% more than the DTJ channel. Moreover, the uniformity of droplet distribution inside the channel was enhanced by 3–20% compared to the DTJ channel geometry.

## Figures and Tables

**Table 1:** Comparison between experimental and simulation for both MDTJ and DTJ in several parameters, (for instance) at 3.1 mL/h oil flow rate and 0.3 mL/h water flow rate.

Parameters	Simulation		Experimental	
	MDTJ	DTJ	MDTJ	DTJ
Diameter (μm)	94	102	80 ± 2.5	93 ± 4
Frequency (d/s)	138	111.1	121	101
Distance (μm)	1608	2081	745 ± 40	875 ± 60

**Table 2:** Illustrates a comparison between our work and some previous experimental work close to our channel dimensions.

**Reference**	[39]	[30]	[15]	[19]	Our work	
**Geometry**	DTJ	-	T-junction	DTJ	DTJ	MDTJ
**Material**	PMMA	PMMA	PDMS	PMMA	PMMA	PMMA
**Fabrication tech.**	Lithography, micromold	CO_2_ Laser	Soft lithography	CO_2_ Laser	CO_2_ Laser	CO_2_ Laser
**Ch. Width**	200 μm	255 μm	100 μm	156 μm	159 μm	159 μm
**Cross Section**	Square	Gaussian	Rectangle	Gaussian	Gaussian	Gaussian
**Oil flow rate**	2 mL/h	-	3 mL/h	6 mL/h	6 mL/h	6 mL/h
**Water flow rate**	0.2 mL/h	-	0.3 mL/h	0.3 mL/h	0.3 mL/h	0.3 mL/h
**Droplet Dia.**	90 μm	-	117 μm	60 μm	66 μm	54 μm
**Cost/chip**	>>1$	-	>1$	1$	<30 cents	<30 cents
**Fabric. time**	>1 day	-	>1 hr	10–20 min.	20 min.	20 min.

## References

[1] Cao L., Cui X., Hu J., Li Z., Choi J.R., Yang Q., Lin M., Li Y.H., Xu F. (2017). Advances in digital polymerase chain reaction (dPCR) and its emerging biomedical applications. Biosens. Bioelectron..

[2] Laird P.W. (2010). Principles and challenges of genome-wide DNA methylation analysis. Nat. Rev. Genet..

[3] Fan S., Chi W. (2016). Methods for genome-wide DNA methylation analysis in human cancer. Brief. Funct. Genomics.

[4] Whale A.S., Huggett J.F., Cowen S., Speirs V., Shaw J., Ellison S., Foy C.A., Scott D.J. (2012). Comparison of microfluidic digital PCR and conventional quantitative PCR for measuring copy number variation. Nucleic Acids Res..

[5] Bu M., Perch-Nielsen I.R., Sørensen K.S., Skov J., Sun Y., Bang D.D., Pedersen M.E., Hansen M.F., Wolff A. (2013). A temperature control method for shortening thermal cycling time to achieve rapid polymerase chain reaction (PCR) in a disposable polymer microfluidic device. J. Micromech. Microeng..

[6] Angeli P., Gavriilidis A. (2008). Hydrodynamics of Taylor flow in small channels: A Review. Proc. Inst. Mech. Eng. Part C J. Mech. Eng. Sci..

[7] Raj R., Mathur N., Buwa V.V. (2010). Numerical Simulations of Liquid−Liquid Flows in Microchannels. Ind. Eng. Chem. Res..

[8] Zhang C., Xing D. (2007). Miniaturized PCR chips for nucleic acid amplification and analysis: Latest advances and future trends. Nucleic Acids Res..

[9] Bandara T., Chandrashekar M., Rosengarten G. (2017). Cfd Modelling of Liquid-Liquid Slug Flowheat Transfer in Microchannels. 28th Int. Symp. Transp. Phenom..

[10] Zhang Y., Ozdemir P. (2009). Microfluidic DNA amplification—A review. Anal. Chim. Acta.

[11] Daw R., Finkelstein J. (2006). Lab on a chip. Nature.

[12] Teh S.-Y., Lin R., Hung L.-H., Lee A.P. (2008). Droplet microfluidics. Lab Chip.

[13] Trantidou T., Friddin M.S., Salehi-Reyhani A., Ces O., Elani Y. (2018). Droplet microfluidics for the construction of compartmentalised model membranes. Lab Chip.

[14] Christopher G.F., Anna S.L. (2007). Microfluidic methods for generating continuous droplet streams. J. Phys. D Appl. Phys..

[15] Chakraborty I., Ricouvier J., Yazhgur P., Tabeling P., Leshansky A.M. (2019). Droplet generation at Hele-Shaw microfluidic T-junction. Phys. Fluids.

[16] Muijlwijk K., Berton-Carabin C., Schroën K. (2016). Cross-flow microfluidic emulsification from a food perspective. Trends Food Sci. Technol..

[17] Faustino V., Catarino S.O., Lima R., Minas G. (2016). Biomedical microfluidic devices by using low-cost fabrication techniques: A review. J. Biomech..

[18] Zhang Y., Jiang H.R. (2016). A review on continuous-flow microfluidic PCR in droplets: Advances, challenges and future. Anal. Chim. Acta.

[19] Islam M.M., Loewen A., Allen P.B. (2018). Simple, low-cost fabrication of acrylic based droplet microfluidics and its use to generate DNA-coated particles. Sci. Rep..

[20] Muck A., Wang J., Jacobs M., Chen G., Chatrathi M.P., Jurka V., Výborný Z., Spillman S.D., Sridharan G., Schöning M.J. (2004). Fabrication of poly(methyl methacrylate) microfluidic chips by atmospheric molding. Anal. Chem..

[21] Kim J.A., Lee J.Y., Seong S., Cha S.H., Lee S.H., Kim J.J., Park T.H. (2006). Fabrication and characterization of a PDMS-glass hybrid continuous-flow PCR chip. Biochem. Eng. J..

[22] Greener J., Li W., Rem J., Voicu D., Pakharenko V., Tang T., Kumacheva E. (2010). Rapid, cost-efficient fabrication of microfluidic reactors in thermoplastic polymers by combining photolithography and hot embossing. Lab Chip.

[23] Chiarello E., Gupta A., Mistura G., Sbragaglia M., Pierno M. (2017). Droplet breakup driven by shear thinning solutions in a microfluidic T-junction. Phys. Rev. Fluids.

[24] Nisisako T., Torii T., Higuchi T. (2002). Droplet formation in a microchannel network. Lab Chip.

[25] Liu Z., Xu W., Hou Z., Wu Z. (2016). A Rapid Prototyping Technique for Microfluidics with High Robustness and Flexibility. Micromachines.

[26] Au A.K., Lee W., Folch A. (2014). Mail-order microfluidics: Evaluation of stereolithography for the production of microfluidic devices. Lab Chip.

[27] He Y., Wu Y., Fu J., Gao Q., Qiu J. (2016). Developments of 3D Printing Microfluidics and Applications in Chemistry and Biology: A Review. Electroanalysis.

[28] Torabi K., Farjood E., Hamedani S. (2015). Rapid Prototyping Technologies and their Applications in Prosthodontics, a Review of Literature. J. Dent. (Shiraz, Iran).

[29] Ogilvie I.R.G., Sieben V.G., Floquet C.F.A., Zmijan R., Mowlem M.C., Morgan H. (2010). Reduction of surface roughness for optical quality microfluidic devices in PMMA and COC. J. Micromech. Microeng..

[30] Prakash S., Kumar S. (2015). Fabrication of microchannels on transparent PMMA using CO_2_ Laser (10.6 μm) for microfluidic applications: An experimental investigation. Int. J. Precis. Eng. Manuf..

[31] Pal P., Sato K. (2009). Various shapes of silicon freestanding microfluidic channels and microstructures in one-step lithography. J. Micromech. Microeng..

[32] Cheng Y., Sugioka K., Midorikawa K., Masuda M., Toyoda K., Kawachi M., Shihoyama K. (2003). Control of the cross-sectional shape of a hollow microchannel embedded in photostructurable glass by use of a femtosecond laser. Opt. Lett..

[33] Osellame R., Maselli V., Vazquez R.M., Ramponi R., Cerullo G. (2007). Integration of optical waveguides and microfluidic channels both fabricated by femtosecond laser irradiation. Appl. Phys. Lett..

[34] Nieto D., Couceiro R., Aymerich M., Lopez-Lopez R., Abal M., Flores-Arias M.T. (2015). A laser-based technology for fabricating a soda-lime glass based microfluidic device for circulating tumour cell capture. Colloids Surf. B.

[35] Lim D., Kamotani Y., Cho B., Mazumder J., Takayama S. (2003). Fabrication of microfluidic mixers and artificial vasculatures using a high-brightness diode-pumped Nd:YAG laser direct write method. Lab Chip.

[36] Liu H.-B., Gong H.-Q. (2009). Templateless prototyping of polydimethylsiloxane microfluidic structures using a pulsed CO_2_ laser. J. Micromech. Microeng..

[37] Day D., Gu M. (2005). Microchannel fabrication in PMMA based on localized heating by nanojoule high repetition rate femtosecond pulses. Opt. Express.

[38] Wang S.-C., Lee C.-Y., Chen H.-P. (2006). Thermoplastic microchannel fabrication using carbon dioxide laser ablation. J. Chromatogr. A.

[39] Yu H., Chong Z.Z., Tor S.B., Liu R., Loh N.H. (2015). Low temperature and deformation-free bonding of PMMA microfluidic devices with stable hydrophilicity via oxygen plasma treatment and PVA coating. RSC Adv..

[40] Chen X., Li T., Shen J. (2016). CO_2_ Laser Ablation of Microchannel on PMMA Substrate for Effective Fabrication of Microfluidic Chips. Int. Polym. Process..

[41] Maselli V., Osellame R., Cerullo G., Ramponi R., Laporta P. (2006). Fabrication of long microchannels with circular cross section using astigmatically shaped femtosecond laser pulses and chemical etching. Appl. Phys. Lett..

[42] Hnatovsky C., Taylor R.S., Simova E., Rajeev P.P., Rayner D.M., Bhardwaj V.R., Corkum P.B. (2006). Fabrication of microchannels in glass using focused femtosecond laser radiation and selective chemical etching. Appl. Phys. A.

[43] Prakash S., Kumar S. (2017). Fabrication of rectangular cross-sectional microchannels on PMMA with a CO_2_ laser and underwater fabricated copper mask. Opt. Laser Technol..

[44] Helmy M.O., Fath El-Bab A.M., El-Hofy H.A. (2015). Elimination of Clogging in PMMA Microchannels Using Water Assisted CO_2_ Laser Micromachining. Appl. Mech. Mater..

[45] Helmy M.O., Fath El-Bab A.R., El-Hofy H.A. (2018). Fabrication and characterization of polymethyl methacrylate microchannel using dry and underwater CO_2_ laser. Proc. Inst. Mech. Eng. Part N J. Nanomater. Nanoeng. Nanosyst..

[46] Imran M., Rahman R.A., Ahmad M., Akhtar M.N., Usman A., Sattar A. (2016). Fabrication of microchannels on PMMA using a low power CO_2_ laser. Laser Phys..

[47] Bhuyan M.K., Courvoisier F., Lacourt P.-A., Jacquot M., Furfaro L., Withford M.J., Dudley J.M. (2010). High aspect ratio taper-free microchannel fabrication using femtosecond Bessel beams. Opt. Express.

[48] Brown L., Koerner T., Horton J.H., Oleschuk R.D. (2006). Fabrication and characterization of poly(methylmethacrylate) microfluidic devices bonded using surface modifications and solvents. Lab Chip.

[49] Tsao C.-W., DeVoe D.L. (2009). Bonding of thermoplastic polymer microfluidics. Microfluid. Nanofluid..

[50] Kelly R.T., Woolley A.T. (2003). Thermal bonding of polymeric capillary electrophoresis microdevices in water. Anal. Chem..

[51] DU L., Chang H., Song M., Liu C. (2012). The effect of injection molding PMMA microfluidic chips thickness uniformity on the thermal bonding ratio of chips. Microsyst. Technol..

[52] Abdel Nasser G., Fath El-Bab A.M.R., Mohamed H., Abouelsoud A. Low Cost Micro-Droplet Formation Chip with a Hand-Operated Suction Syringe. Proceedings of the 2018 IEEE 18th International Conference on Bioinformatics and Bioengineering (BIBE).

[53] Gu Z., Liow J.L. Micro-droplet formation with non-Newtonian solutions in microfluidic T-junctions with different inlet angles. Proceedings of the 2012 7th IEEE International Conference on Nano/Micro Engineered and Molecular Systems (NEMS).

[54] Thorsen T., Roberts R.W., Arnold F.H., Quake S.R. (2001). Dynamic Pattern Formation in a Vesicle-Generating Microfluidic Device. Phys. Rev. Lett..

[55] Ngo I.-L., Woo Joo S., Byon C. (2016). Effects of Junction Angle and Viscosity Ratio on Droplet Formation in Microfluidic Cross-Junction. J. Fluids Eng..

[56] Yu W., Liu X., Zhao Y., Chen Y. (2019). Droplet generation hydrodynamics in the microfluidic cross-junction with different junction angles. Chem. Eng. Sci..

[57] Jin B.-J., Yoo J.Y. (2012). Visualization of droplet merging in microchannels using micro-PIV. Exp. Fluids.

[58] Diehl F., Li M., He Y., Kinzler K.W., Vogelstein B., Dressman D. (2006). BEAMing: Single-molecule PCR on microparticles in water-in-oil emulsions. Nat. Methods.

[59] Rosenfeld L., Lin T., Derda R., Tang S.K.Y. (2014). Review and analysis of performance metrics of droplet microfluidics systems. Microfluid. Nanofluid..

[60] Nekouei M., Vanapalli S.A. (2017). Volume-of-fluid simulations in microfluidic T-junction devices: Influence of viscosity ratio on droplet size. Phys. Fluids.

[61] Osher S., Sethian J.A. (1988). Fronts propagating with curvature-dependent speed: Algorithms based on Hamilton-Jacobi formulations. J. Comput. Phys..

[62] Brackbill J., Kothe D., Zemach C. (1992). A continuum method for modeling surface tension. J. Comput. Phys..

[63] Hirt C., Nichols B. (1981). Volume of fluid (VOF) method for the dynamics of free boundaries. J. Comput. Phys..

[64] Garstecki P., Fuerstman M.J., Stone H.A., Whitesides G.M. (2006). Formation of droplets and bubbles in a microfluidic T-junction—scaling and mechanism of break-up. Lab Chip.

[65] Chinaud M., Roumpea E.-P., Angeli P. (2015). Studies of plug formation in microchannel liquid–liquid flows using advanced particle image velocimetry techniques. Exp. Therm. Fluid Sci..

[66] Lin C.H., Chao C.H., Lan C.W. (2007). Low azeotropic solvent for bonding of PMMA microfluidic devices. Sens. Actuators B.

[67] Lankveld J.M., Lyklema J. (1972). Adsorption of polyvinyl alcohol on the paraffin—water interface. I. Interfacial tension as a function of time and concentration. J. Colloid Interface Sci..

